# Decentration and tilt of plate-haptic multifocal intraocular lenses in myopic eyes

**DOI:** 10.1186/s40662-020-00186-3

**Published:** 2020-04-07

**Authors:** Jiaqi Meng, Wenwen He, Xianfang Rong, Ao Miao, Yi Lu, Xiangjia Zhu

**Affiliations:** 1 Eye Institute, Eye and ENT Hospital of Fudan University, 83 Fenyang Road, Shanghai, 200031 China; 2 Key Laboratory of Myopia, Ministry of Health, Shanghai, China; 3 Key Laboratory of Visual Impairment and Restoration, Shanghai, China; 4 Key NHC key Laboratory of Myopia (Fudan University), Shanghai, China; 5 Laboratory of Myopia, Chinese Academy of Medical Sciences, Shanghai, China

**Keywords:** Decentration, Tilt, Multifocal intraocular lens, High myopia, Cataract surgery

## Abstract

**Background:**

To investigate the decentration and tilt of plate-haptic multifocal intraocular lenses (MfIOLs) in myopic eyes.

**Methods:**

Myopic (axial length [AXL] > 24.5 mm) and non-myopic (21.0 mm < AXL ≤ 24.5 mm) cataract eyes were enrolled in this prospective study and randomly assigned to receive implantation of Zeiss AT LISA tri 839MP lenses (Group A) or Tecnis ZMB00 lenses (Group B). In total, 122 eyes of 122 patients were available for analysis. Decentration and tilt of MfIOLs, high-order aberrations (HOAs), and modulation transfer functions (MTFs) were evaluated using the OPD-Scan III aberrometer 3 months postoperatively. Subjective symptoms were assessed with a Quality of Vision questionnaire.

**Results:**

Near and distance visual acuities, tilt and horizontal decentration did not differ between the two groups, postoperatively. However, myopic eyes of Group B showed greater vertical decentration than those of Group A (− 0.17 ± 0.14 mm vs. -0.03 ± 0.09 mm, respectively), particularly when the MfIOLs were placed horizontally or obliquely. Overall decentration of myopic eyes was greater in Group B than in Group A (0.41 ± 0.15 mm vs. 0.16 ± 0.10 mm, respectively). In Group B, AXL was negatively correlated with vertical decentration and positively correlated with overall decentration. No such correlations were found in Group A. Intraocular total HOAs, coma, trefoil and spherical aberrations were lower in Group A than in Group B for a 6.0 mm pupil among myopic eyes. Generally, Group A had better MTFs and fewer subjective symptoms than Group B among myopic eyes.

**Conclusions:**

Plate-haptic design of MfIOLs may be a suggested option for myopic cataract eyes due to the less inferior decentration and better visual quality postoperatively.

## Background

Driven by the increasing expectations and visual demands of cataract patients, cataract surgery has now developed into a form of refractive surgery [1, 2], with multifocal intraocular lenses (MfIOLs) being widely used to reduce patients’ dependence on glasses, for both distance and near vision [3, 4].

Despite increased use, the implantation of MfIOLs in myopic cataract eyes remains controversial. In addition to retinal abnormalities [5], the compatibility between IOLs and capsular bag sizes is an issue that cannot be ignored [6, 7]. In our previous studies, we found that the compatibility between C-loop haptic IOLs and capsular bag sizes tends to decrease with the elongation of axial length (AXL). More specifically, in myopic eyes, C-loop haptic toric IOLs were more likely to rotate [6] or C-loop haptic MfIOLs showed more inferior decentration [7], both of which lead to reduced visual outcomes. We then questioned whether there is a design of MfIOL better suited for the larger capsular bag of myopic eyes.

Recently, we observed that the plate-haptic MfIOLs appeared to show better stability in myopic eyes, in that they seldom spin or migrate to one corner of the capsular bag after gentle manipulation with the nucleus chopper during surgery. Unlike the C-loop haptic design with a gap between the haptics and the optic, the plate-haptic design of MfIOLs achieves direct support from the capsular bag through the four corners of the IOL. Thus, we speculated that this design may present better capsular stability than the C-loop haptic MfIOLs, providing a better option for spectacle independence among myopic eyes undergoing phacoemulsification and IOL implantation.

The aim of the current study was to compare the capsular stability outcomes of plate-haptic and C-loop haptic MfIOLs in myopic eyes by evaluating the decentration and tilt with the OPD-Scan III aberrometer (Nidek Co, Ltd., Gamagori, Japan). Visual quality and patient symptoms were also compared.

## Methods

### Study design

Patients undergoing lens phacoemulsification of cataract and IOL implantation were enrolled in this prospective, randomized, controlled study over a period of 2 years. The study was conducted in accordance with the ethical principles originating from the Declaration of Helsinki and its amendments, consistent with Good Clinical Practices and local regulatory requirements. Written informed consent was obtained from all study subjects prior to enrollment, and the protocols were reviewed and approved by the institutional review board of the Eye and Ear, Nose, and Throat (EENT) Hospital of Fudan University, Shanghai, China, where the study was conducted. The study was affiliated to Shanghai High Myopia Study (registered at www.clinicaltrials.gov, accession number NCT03062085).

### Patient selection

Patients were included in the study if they had cataract with corneal astigmatism < 1.0 diopter (D) and kappa angle < 0.4 mm. Patients were excluded if they had diabetes or if their eyes had zonular weakness, strabismus, retinal pathology, uveitis, glaucoma, or previous intraocular procedures or trauma. During February 1, 2017, and February 1, 2019, at the EENT Hospital of Fudan University, 68 myopic (AXL > 24.5 mm) and 62 non-myopic (21.0 mm < AXL ≤ 24.5 mm) cataract eyes were enrolled and randomly assigned to receive implantation of the Zeiss AT LISA tri 839MP lens (Carl Zeiss Meditec, Jena, Germany; Group A) or the Tecnis ZMB00 lens (Abbott Medical Optics, Santa Ana, California, USA; Group B) according to the random number table. Eyes with severe intraoperative or postoperative complications or lost to follow-up were excluded from data analysis. A total of 122 eyes of 122 patients completed the study and were available for analysis (Non-myopic eyes: 30 eyes in Group A and 28 eyes in Group B; Myopic eyes: 33 eyes in Group A and 31 eyes in Group B).

### Preoperative examinations

Prior to surgery, all the patients underwent complete ophthalmic examinations which included assessment of visual acuity, slit lamp examination, corneal topography (Pentacam HR, OCULUS Optikgerate, Wetzlar, Germany), AXL measurements (IOLMaster700, Carl Zeiss AG, Oberkochen, Germany), fundoscopy, and B-scan ultrasonography.

### Surgical procedure

All the surgeries were performed by a single, experienced surgeon (Prof. Y.L.) using a standard procedure. A 2.6 mm clear corneal incision was made temporally before a 5.5 mm continuous curvilinear capsulorhexis, hydrodissection and phacoemulsification. The IOL was implanted in the capsular bag and adjusted to the center. After thorough removal of the viscoelastic, the incision was hydrated. Following surgery, all patients received topical prednisolone acetate (Allergan Pharmaceutical Ireland, Westport, Ireland), levofloxacin (Cravit, Santen Pharmaceutical), and pranoprofen eye-drops (Pranopulin, Senju Pharmaceutical, Osaka, Japan).

### Postoperative follow-up

Three months after surgery, all patients underwent complete ophthalmic examinations. Uncorrected distance visual acuities (UDVA; logarithm of the minimal angle of resolution, logMAR), corrected distance visual acuities (CDVA; logMAR), and uncorrected near visual acuities (UNVA; logMAR) were assessed.

**Fig. 1:**
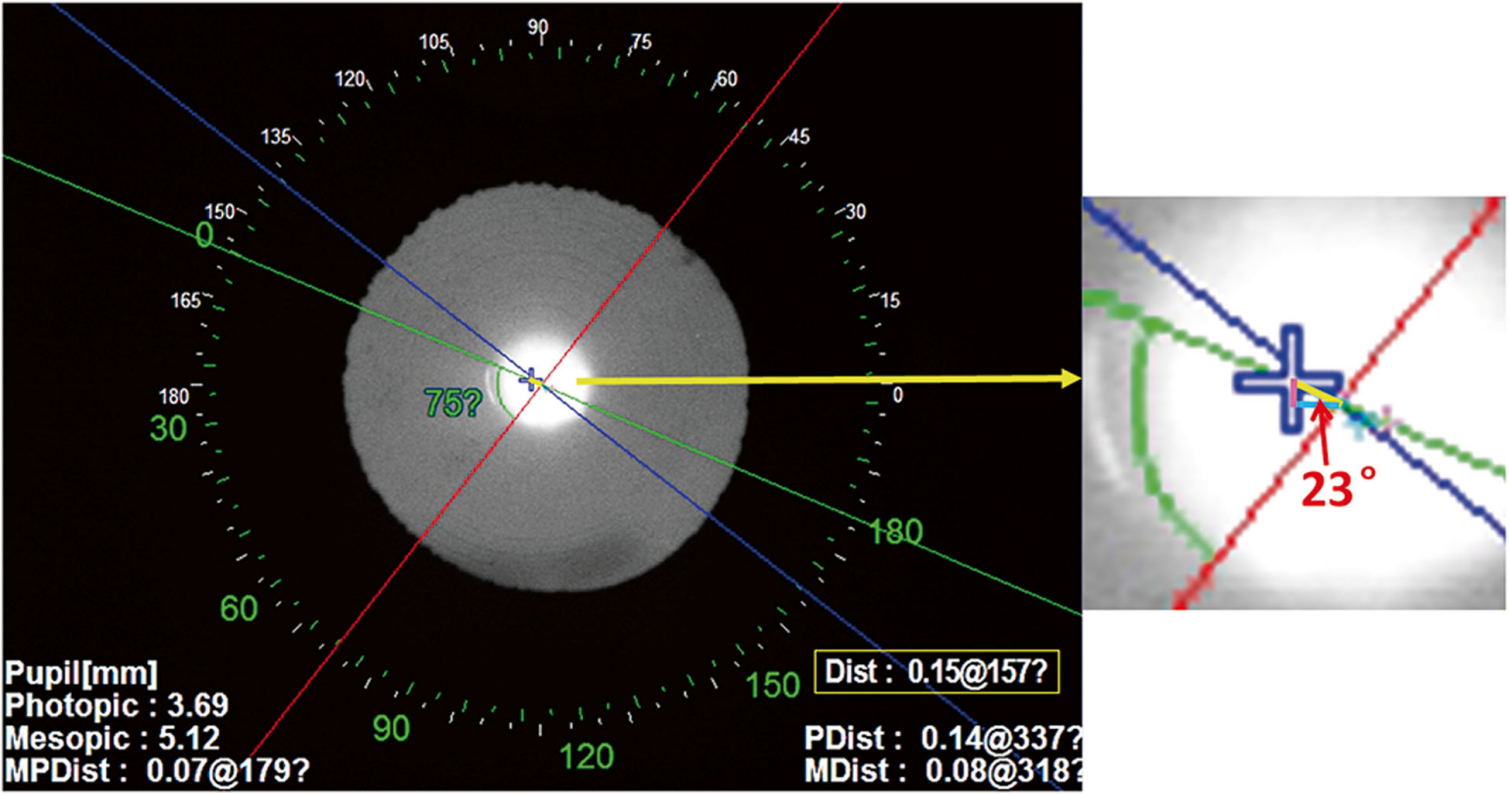
Method for measuring decentration of multifocal intraocular lenses with OPD-scan III aberrometer. (Left) In the retroillumination analysis mode, the center of the visual axis refers to the point of intersection between the red and blue lines. The center of the intraocular lens is indicated by the blue cross. Linking the centers of the visual axis and the intraocular lens, the green line is used to measure overall decentration. The short yellow line crossing the green line represents overall decentration and its direction and length are shown in the yellow box. (Right) The short pink line represents vertical decentration; the light blue line indicates horizontal decentration

The tilt of the MfIOLs was obtained directly from the intraocular tilt data in the wavefront mode of the OPD-Scan III aberrometer, and decentration of the MfIOLs was measured using the same methodology as reported in our previous study [7]. OPD-Scan III assessed the overall decentration as the distance between the centers of MfIOLs and the visual axis in the retroillumination analysis mode. Horizontal and vertical decentration were then determined (Fig. 1). The IOL axis was also recorded under the retroillumination mode. For the plate-haptic IOL, the IOL axis was determined as the line connecting the centers of its two haptics. For the C-loop haptic IOL, the IOL axis was determined as the line linking the two distal points of its haptics. Eyes were assigned to three types of in-bag placements according to the IOL axis (Vertical: IOL axis = 90°; Horizontal: IOL axis = 0°; Oblique: 0° < IOL axis < 90° or 90° < IOL axis < 180°). Ocular and intraocular higher-order aberrations (HOAs) and modulation transfer functions (MTFs) were measured following pupil dilation with a mixture of 0.5% phenylephrine and 0.5% tropicamide (Mydrin-P; Santen Pharmaceutical). The root mean square (RMS) values for the aberrations and MTF data were calculated and recorded for 6.0 mm and 4.0 mm pupil diameters, respectively.

Dysphotopsia symptoms were assessed by a validated Rasch-adjusted Quality of Vision (QoV) questionnaire which included 10 dysphotopsia items [8]. The questionnaire measures 3 aspects of quality of vision: frequency, severity, and bothersome nature of symptoms, including haloes, glare and double vision. Higher QoV scores indicated worse visual quality.

### Statistical analysis

A sample size of 22 per group was calculated to detect a 0.15 mm between-group difference in IOL decentration with an intended power of 90% and a significance level of 5%. A minimum size of 30 patients was enrolled in each group, assuming that some would be lost to follow-up. All continuous data were presented as mean ± standard deviation. Between-group differences for continuous data were assessed using Student’s t-test, and categorical variables were compared using the χ^2^ test. Pearson’s correlation analyses were used to analyze relationships between continuous variables. All *P* values were 2-sided, and P values less than 0.05 were considered statistically significant. Statistical analysis was performed using SPSS version 22 (SPSS, Chicago, Illinois, USA).

## Results

### Patient characteristics

Characteristics of all study patients are shown in Table 1. The mean AXL in the myopic group was 26.45 ± 1.25 mm, with a range between 24.52 mm and 29.07 mm. No statistically significant differences were found in age, sex, operated eye, AXL, kappa angle and preoperative UDVA between the two MfIOL groups (Student’s t-tests for age, AXL, kappa angle and preoperative visual acuity, χ^2^ tests for sex and operated eye, all *P* > 0.05). Postoperative UDVA, CDVA and UNVA did not demonstrate significant differences between the two groups (Student’s t-tests, all *P* > 0.05).

**Table 1:** Patient characteristics

	Group A (*n* = 63)	Group B (*n* = 59)	*P* value
Age (years)*	60.65 ± 8.33	60.98 ± 11.41	0.855
Sex (male/female)^†^	29/34	25/34	0.684
Eye (right/left)^†^	35/28	26/33	0.205
Axial length (mm)*	24.89 ± 2.08	24.93 ± 1.78	0.906
Kappa angle (mm)*	0.21 ± 0.10	0.22 ± 0.10	0.559
Pre-UDVA (logMAR)*	0.63 ± 0.26	0.60 ± 0.28	0.496
Post-UDVA (logMAR)*	0.09 ± 0.14	0.09 ± 0.10	0.953
Post-CDVA (logMAR)*	0.03 ± 0.13	0.03 ± 0.08	0.670
Post-UNVA (logMAR)*	0.05 ± 0.13	0.07 ± 0.09	0.507

*UDVA* = Uncorrected distance visual acuity; *logMAR* = Logarithm of the minimal angle of resolution; *CDVA* = Corrected distance visual acuity; *UNVA* = Uncorrected near visual acuity

Data are mean ± standard deviation. *P* values < 0.05 were considered statistically significant. * Student’s t-test, ^†^ χ^2^ test

### Postoperative decentration and tilt

There were no significant differences between the two groups for tilt and horizontal decentration (Student’s t-tests, both *P* > 0.05). Group B presented significantly greater vertical and overall decentration than Group A (Group B vs. Group A: vertical: − 0.12 ± 0.15 mm vs. -0.03 ± 0.08 mm, respectively, Student’s t-test, *P* <  0.001; overall: 0.31 ± 0.17 mm vs. 0.15 ± 0.09 mm, respectively, Student’s t-test, P <  0.001). The comparisons of the two MfIOLs among non-myopic and myopic eyes in terms of the postoperative decentration and tilt are shown in Table 2. Myopic eyes in Group B presented significantly greater vertical and overall decentration than those of Group A (Student’s t-tests, both *P* < 0.001), while no such differences were found for horizontal decentration and tilt either among myopic or non-myopic eyes (Student’s t-tests, all *P* > 0.05).

**Table 2:** Postoperative decentration and tilt among non-myopic and myopic eyes

Decentration and tilt	Non-Myopic Axial length ≤ 24.5 mm			Myopic Axial length > 24.5 mm		
	Group A (*n* = 30)	Group B (*n* = 28)	*P* value*	Group A (*n* = 33)	Group B (*n* = 31)	*P* value*
Overall decentration (mm)	0.14 ± 0.08	0.19 ± 0.11	0.060	0.16 ± 0.10	0.41 ± 0.15	< 0.001
Vertical decentration (mm)	− 0.02 ± 0.08	− 0.06 ± 0.15	0.187	− 0.03 ± 0.09	−0.17 ± 0.14	< 0.001
Horizontal decentration (mm)	0.01 ± 0.12	0.02 ± 0.12	0.764	0.01 ± 0.16	0.03 ± 0.36	0.814
Intraocular tilt (μm)	0.61 ± 0.21	0.66 ± 0.31	0.469	0.54 ± 0.27	0.65 ± 0.36	0.178

Data are mean ± standard deviation

* *P* values < 0.05 were considered statistically significant. Student’s t-test

The influence of MfIOL in-bag placements on vertical decentration are shown in Table 3 and no differences in ratios of three placements between the two groups were identified (χ^2^ tests, *P* = 0.579; *P* = 0.735 and *P* = 0.545 for the non-myopic and myopic eyes, respectively). Among non-myopic eyes, vertical decentration showed no difference between the 2 types of MfIOLs, regardless of IOL placement (Student’s t -tests, all *P* > 0.05). However, in myopic eyes, the C-loop haptic MfIOLs showed significantly greater vertical decentration than the plate-haptic MfIOL when placed horizontally or obliquely (Student’s t-tests, both *P* < 0.05).

**Table 3:** Influence of MfIOL placement on vertical decentration among non-myopic and myopic eyes

Placement	Non-Myopic: Axial length ≤ 24.5 mm					Myopic: Axial length > 24.5 mm				
	Group A (*n* = 30)		Group B (*n* = 28)		*P* value*	Group A (*n* = 33)		Group B (*n* = 31)		*P* value*
	N^a^	Vertical decentration (mm)	N^a^	Vertical decentration (mm)		N^a^	Vertical decentration (mm)	N^a^	Vertical decentration (mm)	
Horizontal	7	−0.03 ± 0.06	6	− 0.13 ± 0.20	0.229	6	− 0.03 ± 0.10	9	−0.22 ± 0.11	0.005
Vertical	5	0.00 ± 0.06	7	−0.02 ± 0.20	0.823	6	−0.03 ± 0.07	6	−0.07 ± 0.13	0.537
Oblique	18	−0.03 ± 0.09	15	− 0.06 ± 0.09	0.289	21	−0.03 ± 0.10	16	−0.17 ± 0.14	0.001

*MfIOL* = Multifocal intraocular lens

Data are mean ± standard deviation

* *P* values < 0.05 were considered statistically significant. Student’s t-test

^a^*N* = Number of eyes

**Fig. 2:**
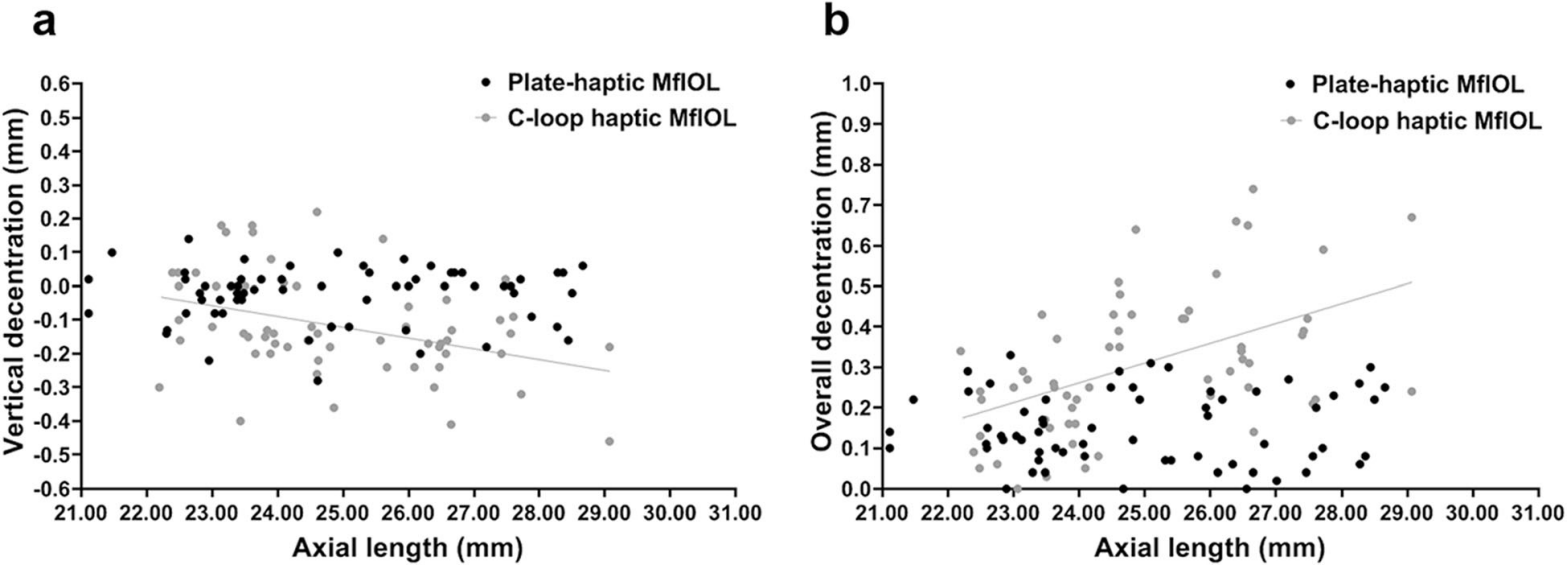
Correlations between decentration of multifocal intraocular lens (MfIOLs) and axial length. **a** The grey line indicates vertical decentration correlated negatively with axial length in Group B (Pearson correlation coefficient, r = − 0.379, *P* = 0.003), while no correlation between vertical decentration and axial length was identified in Group A (Pearson correlation analysis, r = 0.014, *P* = 0.914). **b** The grey line indicates overall decentration correlated positively with axial length in group B (Pearson correlation analysis, r = 0.502, *P* < 0.001), while no correlation between overall decentration and axial length was identified in Group A (Pearson correlation analysis, r = 0.033, *P* = 0.798)

In Group B, AXL was negatively correlated with vertical decentration (Pearson correlation analysis, r = − 0.379, *P* = 0.003; Fig. 2a) and positively correlated with overall decentration (Pearson correlation analysis, r = 0.502, *P* < 0.001; Fig. 2b), while no such correlations were found in Group A (Fig. 2). In both groups, no correlations were found between IOL tilt and AXL, nor between horizontal decentration and AXL (Pearson correlation analyses, all *P* > 0.05).

### Visual quality

**Fig. 3:**
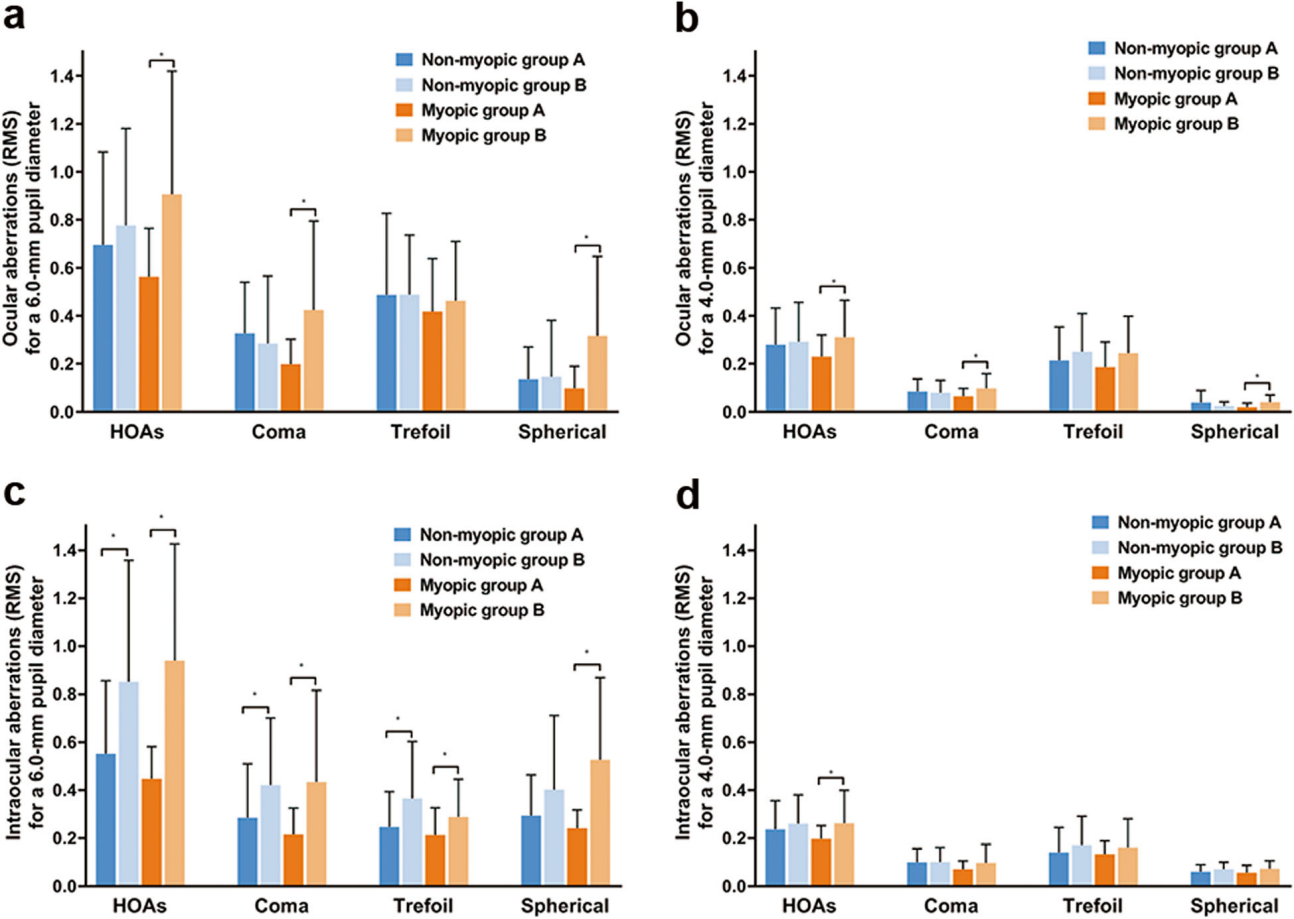
Ocular and Intraocular aberrations. Between-group differences for ocular (**a**, **b**) and intraocular (**c**, **d**) aberrations for a 6.0 mm (**a**; **c**) and 4.0 mm (**b**; **d**) pupil diameter. *A significant difference was found between 2 groups (Student’s t-test, *P* < 0.05). HOAs = higher-order aberrations; RMS = root mean square. Error bars represent standard deviation of the mean

In terms of ocular aberrations, Group A presented significantly lower total HOAs than Group B at 6.0 mm or 4.0 mm pupil diameter (Group A vs. Group B: 6.0 mm pupil: 0.63 ± 0.31 μm vs. 0.85 ± 0.47 μm, respectively, Student’s t-test, *P* = 0.003; 4.0 mm pupil: 0.25 ± 0.13 μm vs. 0.31 ± 0.16 μm, respectively, Student’s t-test, *P* = 0.047). Among myopic eyes, total HOAs, coma and spherical aberrations were significantly lower in Group A than in Group B either for 6.0 mm or 4.0 mm pupil diameter (Student’s t-tests, all *P* < 0.05; Fig. 3a and b). In terms of intraocular aberrations, Group A showed significantly lower total HOAs than Group B either for 6.0 mm or 4.0 mm pupil diameter (Group A vs. Group B: 6.0 mm pupil: 0.50 ± 0.23 μm vs. 0.90 ± 0.49 μm, respectively, Student’s t-test, *P* < 0.001; 4.0 mm pupil: 0.22 ± 0.09 μm vs. 0.26 ± 0.13 μm, respectively, Student’s t-test, *P* = 0.033). Myopic eyes of Group A showed lower total HOAs than those of Group B either for 6.0 mm or 4.0 mm pupil diameter, and lower coma, trefoil and spherical aberrations were also found in myopic eyes of Group A, as compared to those of Group B for a 6.0 mm pupil diameter (Student’s t-tests, all *P* < 0.05; Fig. 3c and d).

**Fig. 4:**
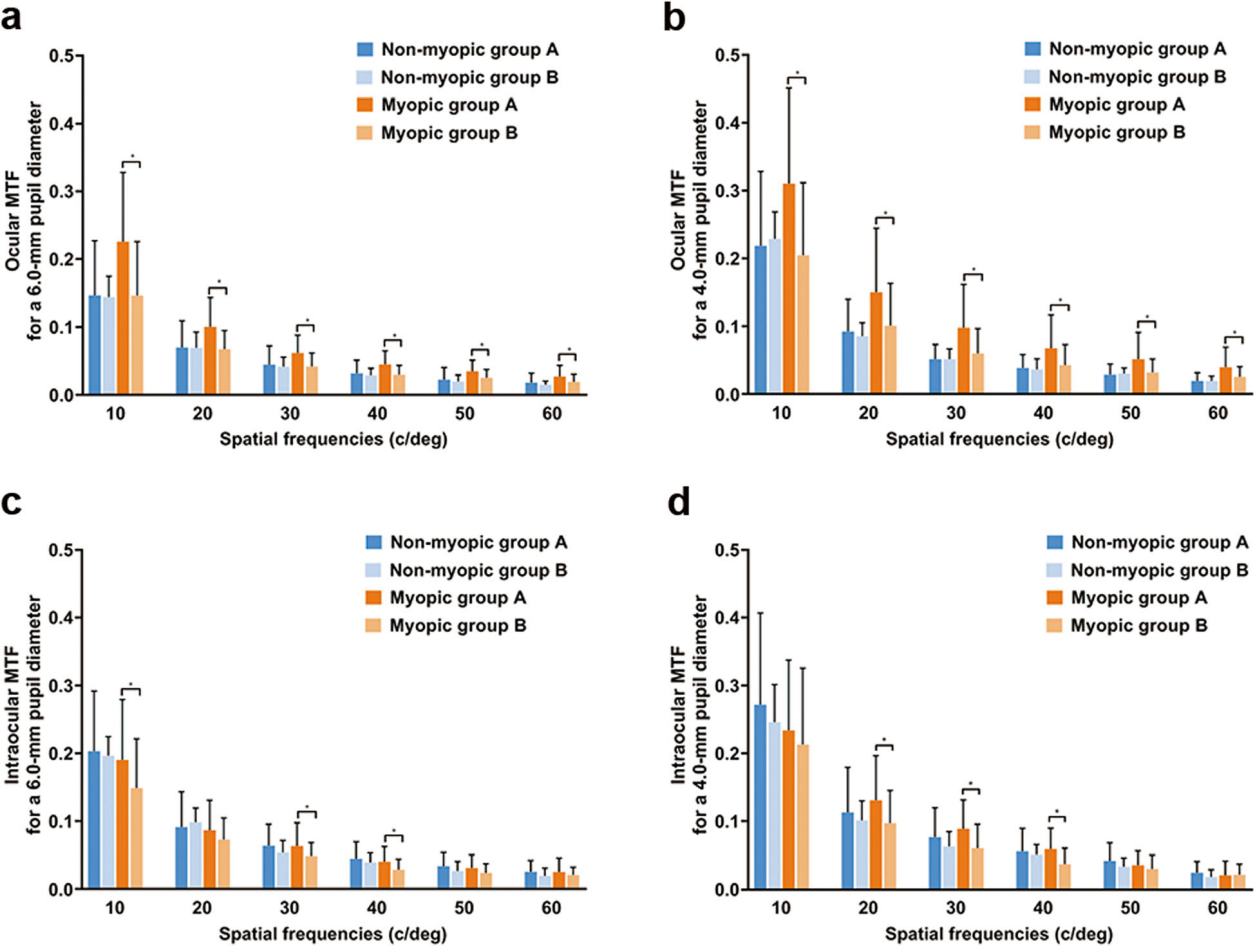
Ocular and intraocular MTFs. Between-group differences in ocular (**a**, **b**) and intraocular (**c**, **d**) MTFs at different spatial frequencies for a 6.0 mm (**a**, **c**) and 4.0 mm (**b**, **d**) pupil diameter *A significant difference was found between 2 groups (Student’s t-test, *P* < 0.05). MTF = modulation transfer function. Error bars represent standard deviation of the mean

Total ocular MTF (area under the curve) was significantly higher in eyes of Group A than those of Group B for a 6.0 mm pupil diameter (Group A vs. Group B: 43.16 ± 11.40% vs. 37.07 ± 11.91%, respectively, Student’s t-test, *P* = 0.005). Myopic eyes of Group A presented a significantly higher total ocular MTF than those of Group B for a 6.0 mm pupil diameter (Group A vs. Group B: 43.10 ± 11.92% vs. 36.72 ± 9.03%, respectively, Student’s t-test, *P* = 0.019). For both the 6.0 mm and 4.0 mm pupil diameter, ocular MTFs were higher in Group A than in Group B, especially among myopic eyes (Fig. 4a and b). In terms of intraocular MTFs, significantly higher MTFs at intermediate spatial frequencies, such as 30 and 40 cpd, were identified in the myopic eyes of Group A as compared to those in Group B for either 6.0 mm or 4.0 mm pupil diameter (Student’s t-tests, all *P* < 0.05; Fig. 4c and d).

In terms of subjective symptoms assessed by the QoV questionnaire, Group A had lower Rasch-adjusted QoV scores for frequency and severity of dysphotopsia symptoms than Group B (Group A vs. Group B: frequency: 4.7 ± 5.1 vs. 7.3 ± 6.9, respectively, Student’s t-test, *P* = 0.017; severity: 3.5 ± 3.7 vs. 5.4 ± 5.3, respectively, Student’s t-test, *P* = 0.022). Comparisons of the two MfIOLs among non-myopic and myopic eyes in terms of QoV questionnaire scores are shown in Table 4. Myopic eyes of Group A presented significantly lower Rasch-adjusted QoV scores for frequency and severity of dysphotopsia symptoms than those of Group B (Student’s t-tests, both *P* < 0.05).

**Table 4:** Quality of Vision questionnaire scores among non-myopic and myopic eyes

Scale	Axial length ≤ 24.5 mm			Axial length > 24.5 mm		
	Group A	Group B	*P* value*	Group A	Group B	P value*
Frequency	4.0 ± 5.2	5.5 ± 5.4	0.293	5.2 ± 5.1	9.0 ± 7.8	0.027
Severity	2.9 ± 3.5	3.6 ± 3.9	0.487	4.0 ± 3.8	7.1 ± 5.8	0.017
Bothersomeness	1.3 ± 2.2	1.9 ± 2.5	0.361	2.4 ± 2.4	3.7 ± 3.7	0.122

Data are mean ± standard deviation

* *P* values < 0.05 were considered statistically significant. Student’s t-test

## Discussion

Though MfIOLs have been widely utilized in order to meet patients’ demands on both near and distance vision [9], the application of MfIOLs in myopic eyes has always been challenging [10]. Malposition of MfIOLs in the capsular bag, such as decentration and tilt, can impair the optical performance of these functional IOLs [11, 12]. In our previous study [7], we found greater inferior decentration of C-loop haptic MfIOLs in the capsular bag and consequently worse visual quality in myopic eyes than in emmetropic eyes. In this study, we proposed that plate-haptic MfIOL might be a better option for myopic eyes. Therefore, we compared the decentration and tilt between plate-haptic and C-loop haptic MfIOLs and found that the former demonstrated less inferior decentration in myopic eyes, which enabled better visual quality after surgery.

The implantation of MfIOLs in myopic eyes is a relatively controversial issue [13, 14]. Risk of retinal complications is an important factor to be considered, as it may affect the overall surgical outcome and the cost-benefit ratio of cataract surgery [15]. For those myopic cataract patients with a strong desire for spectacle independence, MfIOLs may still be recommended for patients with relatively healthy fundus, as alternative refractive surgeries, such as laser in situ keratomileusis or intraocular collamer lens implantation, are no longer feasible under the circumstance of cataract. In recent years, either according to clinical investigations [16] or surgeons’ own intraoperative experiences, it has been noted that the capsular bag of myopic eyes is larger than that of emmetropic eyes in that C-loop haptic IOLs are often found to rotate in the capsular bag during surgery in larger eyes. Thus, the capsular stability of MfIOLs is also worthy of consideration in myopic eyes.

In our previous study [7], we found more inferior decentration of C-loop haptic MfIOLs and consequently, a poorer visual quality in myopic eyes than in non-myopic eyes. However, we did not recommend a satisfactory solution to this problem. Recently, we observed that the plate-haptic MfIOL may be an option for myopic cataract eyes [17]. In our current study, we found less inferior and overall decentration in the plate-haptic MfIOL group than in the C-loop haptic MfIOL group among myopic patients. Meanwhile, decentration in plate-haptic MfIOL group did not increase with AXL, indicating a better capsular stability in longer eyes. With the C-loop design, the large gap between the optic and haptics may lead to less support from the capsular bag when the size of capsular bag increases. Moreover, the friction between the 2 haptics and the larger capsular bag, as the main source of support, is not strong enough to compensate for the gravity of the IOL, particularly when the IOL is horizontally placed. Thus, MfIOLs of this design may “sink” slightly in myopic eyes. However, unlike the C-loop design, the plate-haptic design omits the gap between the optic and haptics and gains greater support from the capsular bag through its 4 corners. This means that the IOL is held tightly by the capsular bag, which better addresses the challenges of gravity at any of the in-bag placements.

In addition to the haptic design, the material and overall diameter of IOLs may also affect the capsular stability of IOLs. David F. Chang found that the C-loop acrylic toric IOL had better rotational stability than the plate-haptic silicone toric IOL, due to the fact that the hydrophobic acrylic material could better adhere to capsules than silicone [18]. However, the plate-haptic IOL used in this study is an acrylic IOL rather than a silicone IOL, with a hydrophobic surface. Regarding IOL size, though the overall diameter of AT LISA tri 839 is 11.0 mm, which is a little smaller than that of ZMB00 [19], the former can gain effective support at its four corners of the haptics while the latter may not get enough effective support because of the large gaps between its optic and haptics.

Moreover, the influence of capsulorhexis and capsular fibrosis on IOL decentration cannot be neglected. Eyes with a severe eccentric capsulorhexis are more likely to have a decentered IOL [20]. Small capsulorhexis may also increase the risk of capsular contraction after surgery, leading to IOL malposition [20, 21]. However, all the surgeries were performed carefully by an experienced surgeon, so capsulorhexis may have little influence on this study. Another factor associated with IOL decentration may be capsular fibrosis. But what this study mainly compared was the capsular stability between the two IOLs instead of that between myopic and non-myopic eyes, so capsular fibrosis may not be a major factor.

Capsular stability of IOLs is crucial to their optical performance [22, 23]. IOL malposition, such as decentration and tilt can lead to increased HOAs, including coma and spherical aberrations, and consequently reduce visual quality [24, 25]. High coma aberration often leads to monocular diplopia [26], and spherical aberration is associated with glare [27]. Meanwhile, the more complicated the optic design of the IOL, the more likely it is to be affected by decentration and tilt [28, 29]. Compared with spherical IOLs, decentration and tilt of aspheric IOLs have a greater effect on the visual quality. As David Madrid-Costa found in a cohort study assessing 4 types of IOLs, for 50% contrast, there was a significant decrease in image quality for the 0.4 mm decentered position as compared to centered position for the aspheric IOLs, while there was no such difference for the spherical IOLs [28]. According to Tamer Tandogan and colleagues, decentration of 1 mm might decrease MTF by less than 10% for monofocal IOLs while decentration of 0.5- 0.75 mm could lead to more significant reduction of MTF for multifocal IOLs [29]. Another study identified that decentration of more than 0.4 mm in MfIOLs significantly reduces the optical performance of MfIOLs [30]. In the current study, we observed overall decentration of 0.41 mm in myopic eyes with C-loop haptic MfIOLs, while overall decentration was only 0.16 mm in myopic eyes with plate-haptic MfIOLs. Accordingly, the plate-haptic MfIOL group presented better visual quality than the C-loop haptic MfIOLs. Therefore, the plate-haptic MfIOL may be a feasible solution for spectacle independence of myopic patients with normal retinas. Moreover, due to the firm compressive forces on the capsular bag through the four corners, myopic eyes implanted with the plate-haptic MfIOL may be also less likely to develop severe capsular contraction after cataract surgery than the C-loop haptic MfIOL of the same material [21, 31].

In this study, the following details also need to be clarified. Though postoperative visual acuities did not differ between the two IOLs in myopic eyes, the tendency of IOL decentration had significant influence on visual quality. Closely correlated with patient satisfaction and quality of life, visual quality is a crucial factor to consider in multifocal IOL implantation [30]. Besides, we were only able to use plate-haptic trifocal IOLs instead of plate-haptic bifocal IOLs due to the supply. As this study mainly focused on the influence of haptic design on the capsular stability of IOLs, the optic design might be a secondary factor. Future studies with plate-haptic bifocal IOLs may be beneficial.

## Conclusion

In conclusion, our study found that the plate-haptic MfIOLs presented less inferior decentration and better visual quality than C-loop haptic MfIOLs in myopic eyes, which may provide a preferred option for myopic cataract patients with a strong desire of spectacle independence.

## Data Availability

Available from the corresponding author on reasonable request.

## Abbreviations

MfIOLs: Multifocal intraocular lenses
AXL: Axial length
EENT: Eye and Ear, Nose, and Throat
UDVA: Uncorrected distance visual acuities
LogMAR: Logarithm of the minimal angle of resolution
CDVA: Corrected distance visual acuities
UNVA: Uncorrected near visual acuities
HOA: High-order aberration
MTF: Modulation transfer functions
RMS: Root mean square
QoV: Quality of Vision
